# Mapping Sub-Antarctic Cushion Plants Using Random Forests to Combine Very High Resolution Satellite Imagery and Terrain Modelling

**DOI:** 10.1371/journal.pone.0072093

**Published:** 2013-08-05

**Authors:** Phillippa K. Bricher, Arko Lucieer, Justine Shaw, Aleks Terauds, Dana M. Bergstrom

**Affiliations:** 1 School of Geography and Environmental Studies, University of Tasmania, Hobart, Tasmania, Australia; 2 Centre for Mined Land Rehabilitation, Sustainable Minerals Institute, the University of Queensland, St Lucia, Queensland, Australia; 3 Australian Antarctic Division, Department of the Environment, Water, Heritage and the Arts, Kingston, Tasmania, Australia; 4 Environmental Decision Group, School of Biological Sciences, the University of Queensland, St Lucia, Queensland, Australia; University of Saskatchewan, Canada

## Abstract

Monitoring changes in the distribution and density of plant species often requires accurate and high-resolution baseline maps of those species. Detecting such change at the landscape scale is often problematic, particularly in remote areas. We examine a new technique to improve accuracy and objectivity in mapping vegetation, combining species distribution modelling and satellite image classification on a remote sub-Antarctic island. In this study, we combine spectral data from very high resolution WorldView-2 satellite imagery and terrain variables from a high resolution digital elevation model to improve mapping accuracy, in both pixel- and object-based classifications. Random forest classification was used to explore the effectiveness of these approaches on mapping the distribution of the critically endangered cushion plant *Azorella* *macquariensis* Orchard (Apiaceae) on sub-Antarctic Macquarie Island. Both pixel- and object-based classifications of the distribution of *Azorella* achieved very high overall validation accuracies (91.6–96.3%, κ = 0.849–0.924). Both two-class and three-class classifications were able to accurately and consistently identify the areas where *Azorella* was absent, indicating that these maps provide a suitable baseline for monitoring expected change in the distribution of the cushion plants. Detecting such change is critical given the threats this species is currently facing under altering environmental conditions. The method presented here has applications to monitoring a range of species, particularly in remote and isolated environments.

## Introduction

There is increasing interest in monitoring landscape-scale changes in the distributions of plants caused by impacts including climate change, species invasions, and management actions [1,2]. Monitoring changes in the distribution of individual species or communities often requires the creation of accurate high-resolution maps. Such maps can be used to monitor responses to environmental changes at regional or landscape scales, and hence complement plot-level studies. The production of these maps is time-consuming and expensive, and extensive research has been directed at improving mapping methods [3,4]. The field of remote sensing has produced a wide range of semi-automated image interpretation methods to improve the repeatability and objectivity of the resulting maps [4]. As part of efforts to improve accuracy, these methods are expanding rapidly on a number of fronts, including the development of object-based image analysis techniques [5], the merging of terrain and spectral data in image classifications (e.g. [6]), the use of texture measures to provide contextual information (e.g. [7–9]), and sophisticated classification algorithms such as random forests (RF) [10–12]. Incorporating environmental variables into satellite image classification (e.g. [6,8,13]) has the potential to improve the classification by pairing structural and disturbance information from the satellite imagery with the potential habitat information for individual species from species distribution modelling.

A major division exists between pixel- and object-based image analysis in remotely sensed image classification techniques. Pixel-based classification retains maximum spatial resolution, but has limited ability to incorporate information from neighbouring pixels; although the inclusion of texture measures provides some contextual information [9]. When applied to very high resolution imagery, the variability of the pixels often results in speckled classification results [5,8]. In contrast, geographic object-based image analysis (GEOBIA) starts by segmenting the image into objects made up of contiguous, spectrally similar pixels and the classification is then applied to the objects rather than the pixels. GEOBIA is becoming increasingly popular as a method for managing very high resolution satellite imagery, because the pixels are often smaller than the individual entities to be mapped [5]. However, it is not always obvious *a priori* which level of analysis would be most appropriate for a given mapping application. Where the pixel size is consistently smaller than the entities being mapped, as often occurs with very high resolution imagery, there are clear advantages to an object-based approach, which are lost when the entities being mapped are typically smaller than an individual pixel [5]. It is less clear which of the two approaches is likely to be most useful for mapping entities that vary in size from smaller than a single pixel to larger than multiple contiguous pixels [14].

To improve the interpretation of such data, researchers have begun using tools from the field of machine learning, including RF classification [12]. RF is an ensemble classifier that builds a forest of classification trees, using a different bootstrapped training sample and randomly selected set of predictor variables for each tree. Unweighted voting is then used to produce an overall prediction for each site in the sample [10,15]. RF has been used to classify vegetation with very high accuracy in a number of mapping applications, including mountain forest communities [16], cropping [11], invasive species [12,17,18], and predicting rare species distributions [12]. It has also been shown to perform well in comparison to decision trees and other ensemble classifiers [11] and is able to capture complex, non-linear interactions among noisy, non-normal predictor variables [12,16]. In addition, RF provides measures of variable importance that can be used for exploratory ecological interpretation [11,12,16].

In this study, we examine the capacity of RF for mapping a sub-Antarctic cushion plant species using pixel- and object-based classification of environmental and spectral variables. Our aim is to map the distribution of a critically endangered cushion plant that is endemic to sub-Antarctic Macquarie Island. *Azorella* *macquariensis* Orchard (APIACEAE) was listed as Critically Endangered under the Environment Protection and Biodiversity Conservation Act 1999, Australia after we discovered widespread dieback in late 2008 [19]. To monitor changes in the distribution of *Azorella* on the island, the first step was to produce a high-accuracy fine-scale map of its distribution at the time the dieback was first discovered and when many of the first wave of dead cushion plants were still largely intact, to approximate the distribution pre-dieback distribution. Investigations into the cause of the dieback are continuing.

To produce the map, we extracted a vegetation index and several texture measures from very high resolution WorldView-2 satellite imagery and derived several environmental variables from a fine-resolution digital elevation model (DEM). We then examined the effect of pixel- and object-based analysis on the accuracy of the image classifications and tested how well these classifications were able to predict both the presence and the degree of *Azorella* cover at the landscape scale.

## Methods

### Study Site

**Figure 1:**
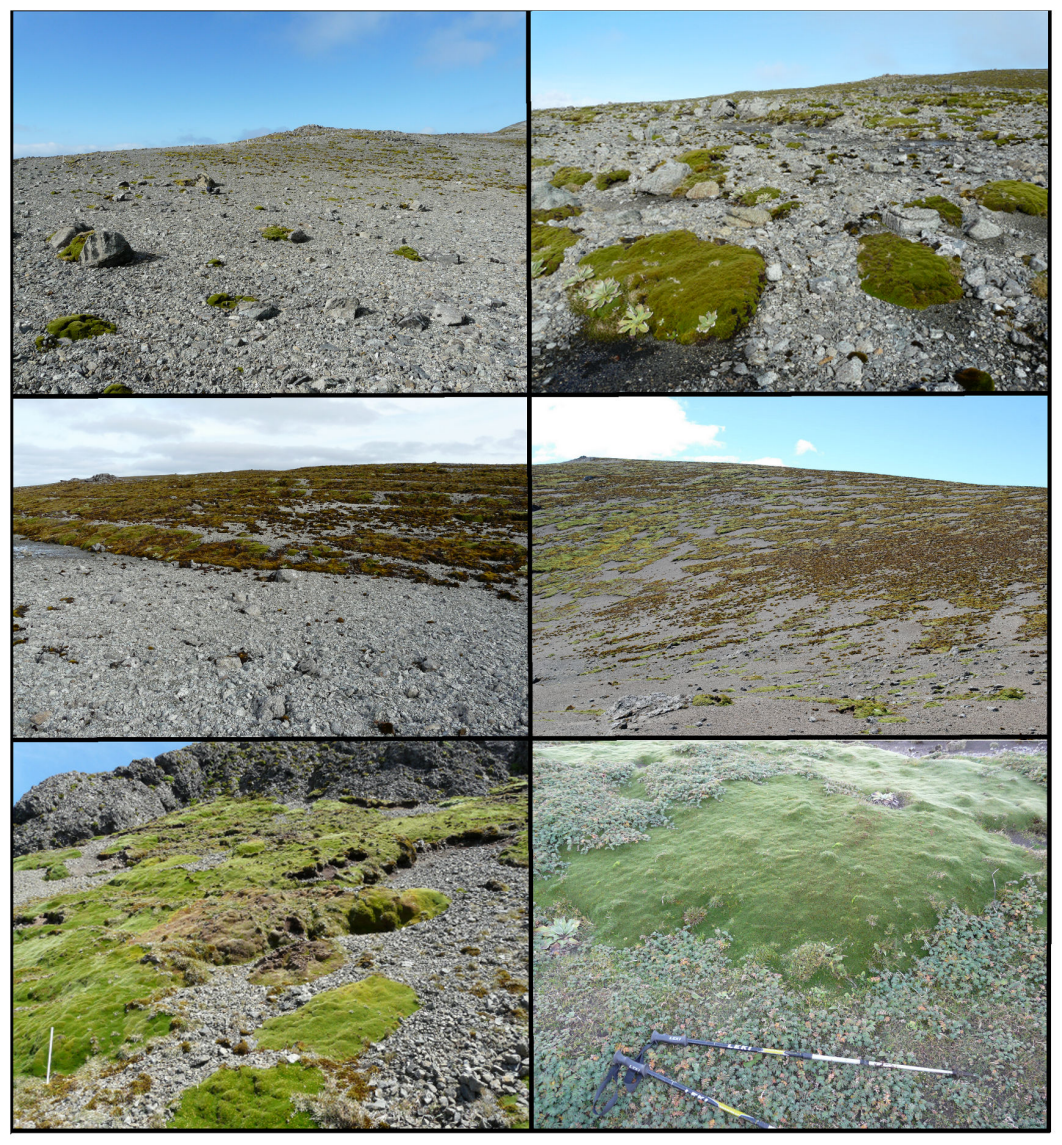
*Azorella* growth patterns. *Azorella* exhibits a range of growth patterns on Macquarie Island, from sparse polar desert (top left) to dense herbfields (bottom right). This variability increases the challenges involved in modelling its distribution.

Macquarie Island (54°30’ S, 158°57’ E) is 12,390 ha in area and dominated by a plateau at 200–400 m altitude, with *Azorella* *macquariensis* occupying the highest parts of the island. The cover of *Azorella* in the landscape is highly variable. It occurs as small cushions on the fringes of mid-altitude plateau grasslands, forms extensive cushions on east-facing higher slopes, with the cushions becoming progressively smaller and sparser in feldmark (vegetation with less than 50% cover) and polar desert zones (Figure 1). In feldmark and polar desert, *Azorella* is usually the dominant vascular plant species, with other species occurring as epiphytes or between cushions. The distribution of cushions tends to be patchy at all spatial scales ranging from tens of centimetres to the entire plateau. There is therefore no ‘natural’ scale of analysis, and both image pixels and objects are likely to contain mixtures of *Azorella* and other species. Thus, the entity being mapped is a patch that contains *Azorella* , and the minimum size of that entity is uncertain and variable.

### Field data

**Figure 2:**
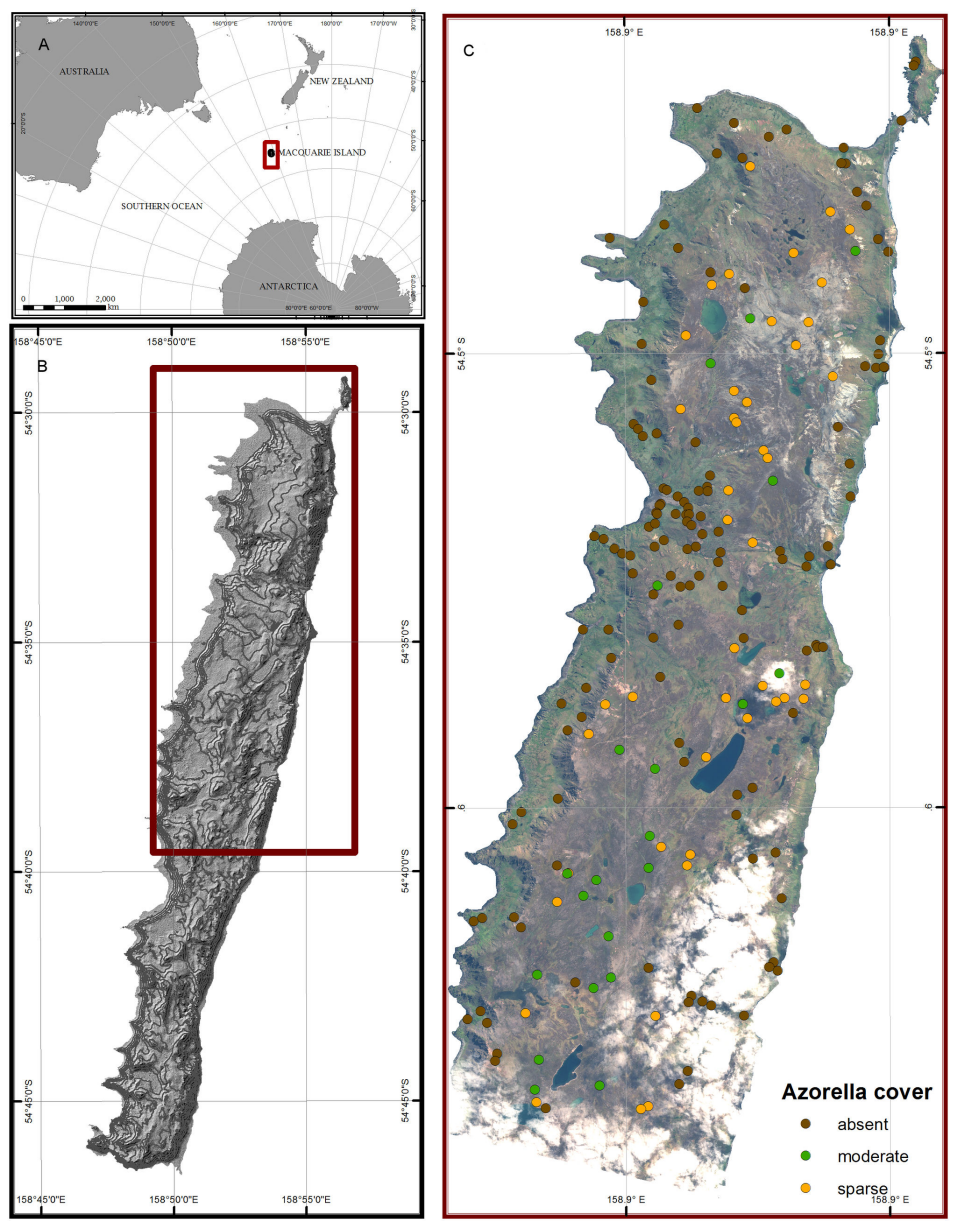
Study area. Field sites on northern Macquarie Island, showing the observed cover class of *Azorella* . Panel (a) shows the location of Macquarie Island in the Southern Ocean; (b) shows the extent of the island; and (c) shows the extent of the WorldView-2 image and location of the training samples. Those field plots in areas obscured by cloud in the image were excluded from the analysis.

Macquarie Island was visited over two summers (November–February) in 2008/9 and in 2009/10, with access granted through permits issued by Tasmania’s Parks and Wildlife Service and the Macquarie Island Research Advisory Committee. During these visits 349 sites were examined, of which 201 were in the cloud-free portion of the available satellite image (Figure 2). Most sites (179) were located using a geographically stratified random sampling design (GeoStrat), with some additional sampling (29 sites) utilising existing sites located in homogeneous patches of plant communities, which are part of an ongoing study [20]. GeoStrat used an unsupervised fuzzy c-means classification of the island based on six terrain variables that were anticipated to affect microclimate (elevation, slope, solar radiation, surface curvature, topographic wetness index, and topographically-deflected mean wind speed) and a normalised difference vegetation index (NDVI) derived from QuickBird satellite imagery.

At each site, a 10 x 10 m plot was laid out and an 8.75 m^2^ (2.5 x 3.5 m) photograph was taken at each corner of the plot (i.e. four photographs per plot) using an elevated camera system with a 14 mm lens suspended 2.6 m above the plots. Point-intercept analysis was used to estimate the cover of all vascular plant species and broad categories of other cover classes, including bryophytes and lichens using Coral ,Point Count software [21] by identifying the cover class underlying 100 randomly located points placed over each photograph. The results were averaged for each plot. *Azorella* occurred in one-third of the plots, ranging from 0.25–26.6% cover at those sites (Table 1). The field plot size was chosen to ensure that the sampled area on the ground represented the pixel at the centre of the plot, regardless of errors in GPS positioning (especially in steep coastal areas where GPS accuracy was reduced) and registration of the image.

**Table 1:** Descriptive statistics of *Azorella* cover in the 200 field sites used to train the image classifications.

**Statistic**	**Present Class**	**Total**
Count	135	201
Mean Cover (%)	4.8	1.5
St Dev Cover (%)	5.5	3.8
Median Cover (%)	2.4	0
Minimum (%)	0.3	0
Maximum (%)	26.6	26.6
Skewness	1.9	3.6
Kurtosis	4.1	15.2

Initial attempts to apply regression models to the cover data proved unsuccessful, as evidenced by a random forest multiple regression where using all input variables only explained 29% of the variance. We subsequently classified the data into two and three classes. The binary classification divided the sites into present (65 sites) and absent (135 sites). The ternary classification divided the sites into absent (135 sites), sparse (44 sites), and moderate cover (21 sites), with the boundary between the sparse and moderate classes set at 5% cover. The binary classification was the primary outcome of this analysis, but if it is possible to accurately distinguish very sparse and moderate cover of *Azorella* , then it will likely be possible to monitor a decrease in *Azorella* cover, as well as contraction of its range. In addition, we believed that it was important to explore the nature of the errors of classification - i.e. whether the strongest separation was between presence/absence or between sparse/moderate cover of *Azorella* .

Due to the reduction in sample size caused by the extensive cloud cover in the available satellite imagery, we were unable to divide the field data into training and test datasets. To validate the classifications, we therefore used data on *Azorella* presence and absence acquired for other ongoing vegetation studies on the island [22]. This dataset comprised 187 randomly located sites, of which 108 contained *Azorella* and 79 did not. These data could not be divided into moderate and sparse classes comparable to the data used for training the ternary classifications.

### Satellite imagery

For northern Macquarie Island, two recent very high resolution satellite images were available for analysis. A cloud-free QuickBird image of the entire island with 2.4 m pixels obtained in March 2005 was used for the GeoStrat sampling design. A mostly cloud-free (88.9%) WorldView-2 image of the northern half of the island, with 2 m multispectral pixels and eight spectral bands, was obtained in December 2009. Atmospheric correction was trialled using the algorithms in ENVI FLAASH; however, the resulting spectra did not meet expectations. This is probably due to poor parameterisation of the atmospheric correction model given unreliable optical thickness parameters. We therefore chose to work with the DN-values of the WorldView-2 image. Given the focus on classification of a single image we did not worry too much about the absolute values. The relative differences between the classes were most important for the classification. From this image, we calculated an NDVI layer and a set of texture measures, which have been shown elsewhere to improve the accuracy of image classification [18,23]. NDVI is a widely used index that captures the relative proportions of red and near infrared (NIR) reflectance in a satellite image, as a proxy for the amount of live vegetation in a pixel [24]. Texture measures provide information about the spatial context of a pixel [23]. Here, we used grey-level co-occurrence matrix (GLCM) texture measures [25,26]. The GLCM was calculated for the NDVI image using an 11 x 11 cell kernel. GLCM computes a matrix that compares the greyscale values of neighbouring cells in a moving window. From the GLCM, we calculated eight texture measures: mean, variance, homogeneity, contrast, dissimilarity, entropy, angular second moment, and correlation [25].

### Terrain data

To explore the controlling environmental parameters influencing the distribution of *Azorella* we incorporated a suite of terrain variables (elevation, wetness, mean topographically-deflected wind speed, curvature, solar radiation, distance from coast, topographic position [27–31]) as proxies for direct environmental variables (see 26) in the analysis (Table 2). We did not include measures of climate variability, despite their popularity in the species distribution modelling literature [33], because Macquarie Island is small and isolated enough to have only a single meteorology station. It is hence not possible to interpolate climate variables across the island, although a lapse rate of around 0.8°C per 100 m altitude has been recorded [34] and this is included by proxy in the elevation data.

**Table 2:** Terrain variables incorporated in the random forest analyses of *Azorella* distribution on Macquarie Island.

**Variable**	**Description**
Elevation	The vegetation on Macquarie Island exhibits strong altitudinal gradients, although elevation only indirectly affects plant physiology [33,42].
Curvature	Spatial data on precipitation were unavailable due to the small size of the island, but surface curvature is known to affect water flow patterns [27].
Wetness index	Topographic wetness index models potential areas of water accumulation. Here, a Monte Carlo simulation with an error term of 5 m and 1000 simulations was used to reduce the effect of DEM errors on the resulting surface [28].
Topographically-deflected wind speed	The prevailing wind on Macquarie Island comes from the west and north west, at a mean speed of 35.1 km/h [51], and we used a topographically-deflected wind speed model to estimate the wind speed across the island [29,30].
Solar radiation	Solar radiation, as the source of energy for photosynthesis, is likely to have a direct impact on vegetation distributions. This was calculated using the solar radiation function in ArcGIS 9.3, for the period of one year.
Distance from coast	Distance from the coast corresponds with the distributions of several plant species on Macquarie Island, including *Azorella* . To incorporate this, we calculated the surface (not planar) distance from the coastline [44].
Ridgeness/valleyness	*Azorella* is observed to be more common in higher, more exposed sites than in gullies, even at high elevations. We calculated two multi-scale measures of topographic position, namely ridgeness and valleyness, using the multi-scale landform classification algorithm in the LandSerf package [52]. This calculated the proportion of scales at which each cell occurred in a ridge or valley for neighbourhood sizes ranging from 3 x 3 to 49 x 49 cell neighbourhoods.

Elevation values were taken from a 5 m resolution DEM of Macquarie Island derived from Airborne Synthetic Aperture RADAR data acquired in 2000 by the NASA PACRIM Mission 2, with heights accurate to 5 m [35,36]. From this DEM, we derived the slope, aspect, mean topographically-deflected wind speed, solar radiation, topographic wetness index, surface curvature (including planar and profile curvature), ridgeness, valleyness, and distance from the coast variables.

### Random Forest Classification

Random forest classification (RF) was used to predict the presence of *Azorella* on the basis of high resolution terrain and spectral data. In these analyses, only two user-determined variables were required: the number of variables in the random subset at each node, and the number of trees in the forest. Some studies suggest that RF can be insensitive to the first of these (e.g. Liaw and Wiener 2002). We used the *randomForest* package in *R* [15] to construct 5000 trees per classification, and plotted the error rates as a function of the number of trees. As the error rate stabilised by 2000 trees at 0.05 for the absent class and 0.15 for the present class, all the classifications were hence constructed using 2000 trees. The number of input variables for each forest varied, but for all forests, a random subset of three input variables was used to split the data at each node of each tree. When the classes in an RF classification are unbalanced, the error rates are highest in the rarest classes [37]. Although techniques have been suggested to correct for this (e.g. [9,15,39]), we chose not to apply them, as our dataset was only moderately unbalanced. Instead, we chose to use the predictive accuracy for the less common ‘present’ class as the primary measure of model performance.

RF produces multiple outputs to aid in interpreting the results. In addition to the class predictions, it calculates the probability of membership for each class, out-of-bag (OOB) accuracy estimates, variable importance measures, and partial dependence plots. OOB errors are calculated in random forest classifications as an alternative to cross-validation. For each tree in the forest, a random third of all observations are held out from the training set, and are referred to as "out-of-bag". The OOB error is, thus, the proportion of these observations that is misclassified. To calculate the variable importance, the mean decrease in accuracy is calculated according to the increase in prediction error when OOB data (cases left out of the bootstrap sample) for that variable are permuted and all other variables are left unchanged [15]. We used partial dependence plots to show the relationships between individual predictor variables and the predicted probability of the presence of *Azorella* and the variable importance measures to guide the selection of variables for inclusion in the final classifications.

### Improving the classification

Three analytical approaches to improve the accuracy of RF classification for mapping the distribution of *Azorella* were assessed:

1 Pixel- versus object-based image analysis
2 A hypothesis-driven subset of available input variables versus a subset of input variables selected according to the random forest variable importance measures
3 The number of classes: a two-class (binary) classification versus a three-class (ternary) classification (presence-absence versus moderate-sparse-absent)

A total of 27 input variables were available for the classifications, including 10 terrain variables and 17 spectral variables (eight spectral bands, eight texture measures, and an NDVI layer). To choose the subset of variables on statistical grounds, a forest was first built using all available input variables. The variable importance plots were used to indicate which variables contributed most to the classification. A heuristic approach was applied to find a minimum set of input variables that maintained the accuracy of the full-model classification. This process showed that including variables with lower importance often decreased the model accuracy by introducing noise. In addition to the improvement of model accuracy, reduced models simplify ecological interpretation of classifications [37].

For the hypothesis-driven subset of input variables, we selected those most likely to maximise the ecological and spectral separation of *Azorella* from other vegetation on spectral and ecological grounds. These variables were the blue, green, yellow, red edge, and near-infrared 2 spectral bands; NDVI; the mean, homogeneity and entropy GLCM texture measures; and the terrain variables.

The most appropriate scale of analysis can be difficult to discern in advance, especially for a species where individuals range in diameter from a few centimetres to several metres, and which is patchy at multiple spatial scales. We therefore repeated all classifications for both individual pixels and for objects. For the pixel-based modelling approach, we extracted values for each image pixel that intersected with the plot boundaries at each site. This approach captured the variability of spectral values within a plot, at increased risk of erroneously including pixels from outside the plot boundaries. This approach is also vulnerable to spatial autocorrelation, but is commonly used in remote sensing applications [38].

For the object-based classifications, the WorldView-2 image was divided into objects using the multi-resolution segmentation algorithm in eCognition Developer 8 software. This segmentation algorithm divides an image into homogeneous regions by grouping neighbouring pixels based on their Euclidean distance in multivariate attribute space. The size of the objects is determined by the scale parameter, which sets the threshold for homogeneity within each object. The value of the scale parameter is generally determined by trial-and-error, because there are no objective methods available to choose an appropriate value [39]. Here, we used the pixel values in the eight spectral bands of the WV-2 image to identify image objects. We set the scale parameter to 35 and the shape parameter to 0 so that the objects could take any shape. Although most *Azorella* cushions are ovoid in shape, they often grow in dense colonies, interspersed with mosses and grasses, with the clumps forming a wide variety of shapes, from almost circular to long, thin, inter-connected terraces. The objects visible in the satellite imagery are these dense clumps, rather than individual cushions. At this scale, objects were observed to follow the edges of these terraces, and had a mean area of 208.4 m^2^. The mean values for the terrain derivatives, spectral bands, NDVI, and texture measures were calculated for each object. These mean values were then subjected to the same RF classification procedure as the pixels.

As the major purpose of this study was to develop a baseline map for change detection, it was important to test whether these classifications simply identified the high elevation, bare areas of the island or whether they could distinguish these from areas that contain small amounts of *Azorella* . We therefore repeated the classifications using three classes – absent, sparse and moderate cover of *Azorella* – and repeated the classifications.

### Model Validation and Accuracy Assessment

Once the classifications had been finalised, we applied them to all pixels/objects in the image to produce predictive maps. We assessed the classification accuracy using the independent validation samples to determine producer’s and user’s accuracy measures. Producer’s accuracy refers to the probability that a patch on the ground is mapped as the appropriate class, while the user’s accuracy refers to the converse - that a patch on the map exists in the real world. As validation accuracy tends to be lowest for the least common class [9], the primary measure of interest was the producer’s accuracy for the present class in the binary classification. It was not possible to separate the moderate and sparse cover classes in the independent dataset, so we grouped these for the validation. We also used expert knowledge and field photographs to informally assess the accuracy of all maps. Finally we calculated kappa statistics of classification accuracy.

## Results

### Binary classifications

**Figure 3:**
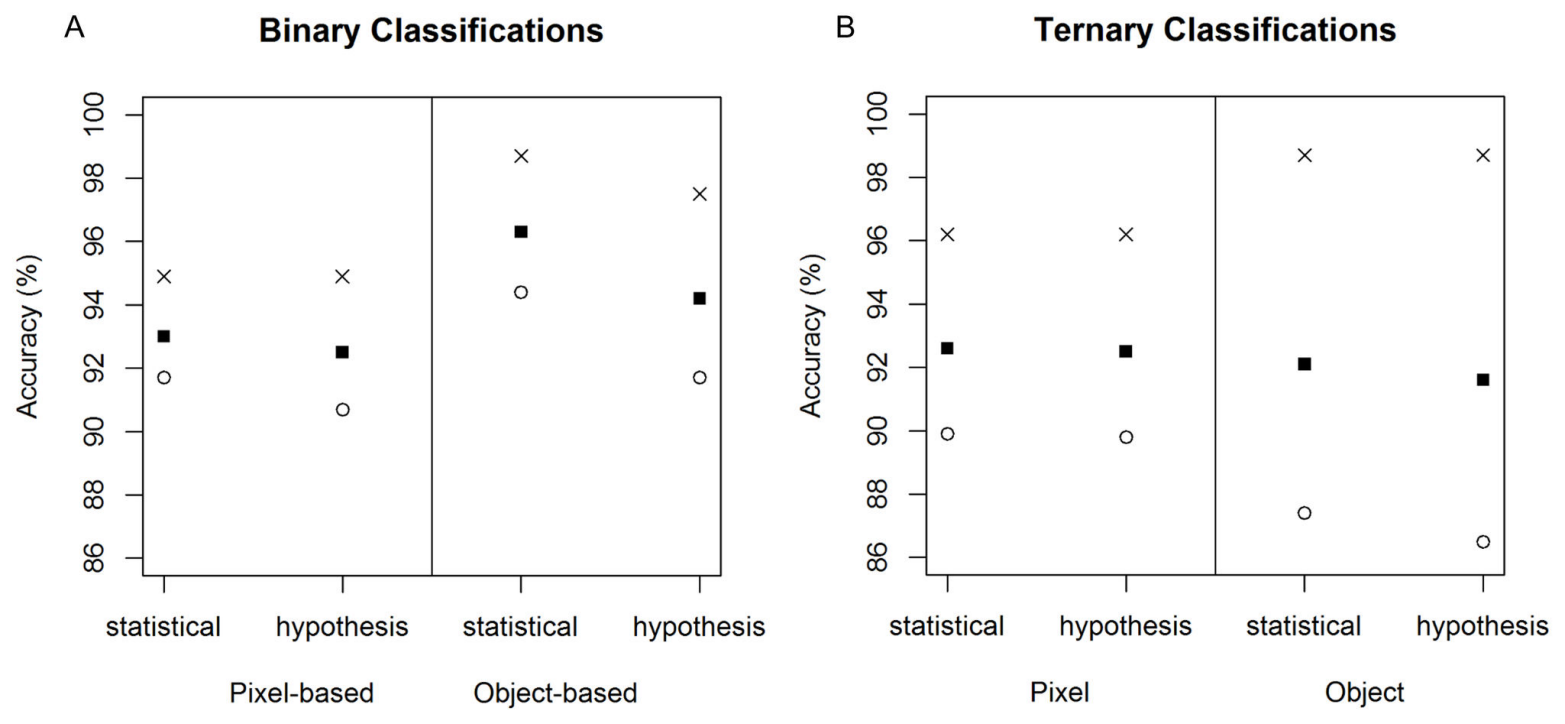
Accuracy of the classifications. The validation producer’s accuracy measures for all classes in all classifications. For the binary classifications (A), object-based models were slightly more accurate than the pixel-based classifications, and the classifications based on statistically-selected subsets of input variables produced slightly higher accuracies than those using a hypothesis-driven subset. For the ternary models (B), the overall accuracies were similar for all models, though they were more variable between classes in the object-based classifications.

Random forest classifications based on a combination of terrain and spectral input variables accurately predicted the presence of *Azorella* with very high accuracy, regardless of which classification was used (κ = 0.848–0.924; Figure 3). There were two major trends in the accuracy scores for the binary classifications: object-based classifications were more accurate than pixel-based classifications, and the statistically-driven subsets of input variables produced more accurate classifications than the hypothesis-driven subsets. Kappa scores showed that the statistically-chosen subset of input variables for the object-based classification was most accurate (κ = 0.924) and the least accurate used a hypothesis-driven subset of variables and pixel-based classification (κ = 0.848). The differences in accuracies were slight, but consistent (Figure 3). Although still high, the accuracy was lowest for the present class in all classifications (90.7–94.4%, compared with 94.9–98.7% for the absent class), probably because it was the smallest class in the training dataset.

**Figure 4:**
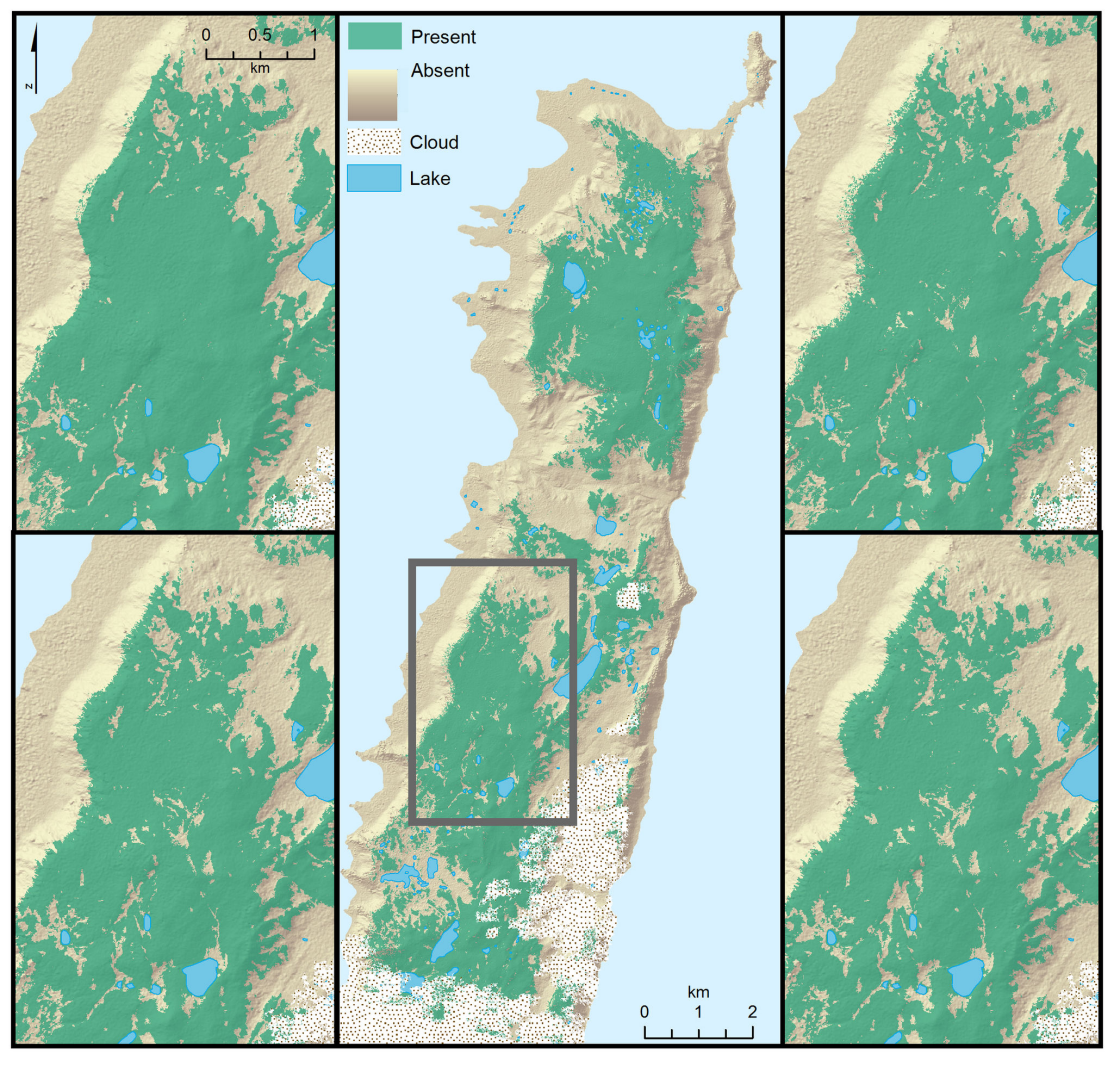
Binary classified map of *Azorella* distribution. Predicted *Azorella* presence on northern Macquarie Island based on binary classifications, showing (A) the entire mapping region, as predicted by hypothesis-driven pixel-based classification. Panels B–D demonstrate the variation in maps in the central part of the mapped area, as predicted by pixel-based classification of a statistically-selected subset of input variables (B); object-based classification of a statistically-selected subset of input variables (C); pixel-based classification of a hypothesis-driven subset of input variables (D); and object-based classification of a hypothesis-driven subset of input variables (E). The stippled area indicates cloud cover.

Differences among the maps produced by all binary classifications were subtle, though the object-based classifications produced a slightly more fragmented pattern of *Azorella* distribution than the pixel-based ones, and the hypothesis-driven subsets of input variables produced slightly more fragmented maps than the statistically-driven subsets (Figure 4). There was obvious spatial structure to the errors in classification, with all misclassifications occurring close to the class boundaries. All validation sites were within 22 m of the class boundary, and all except five were within 10 m of the boundary.

After inspection of the variable importance plots for the binary classifications, the statistically-driven pixel-based classification was based on elevation, solar radiation, coast distance, ridgeness, red edge, NDVI and the GLCM mean texture measure. The object-based classification required six of the same variables, but replaced ridgeness with the NIR 1 and NIR 2 spectral bands.

**Figure 5:**
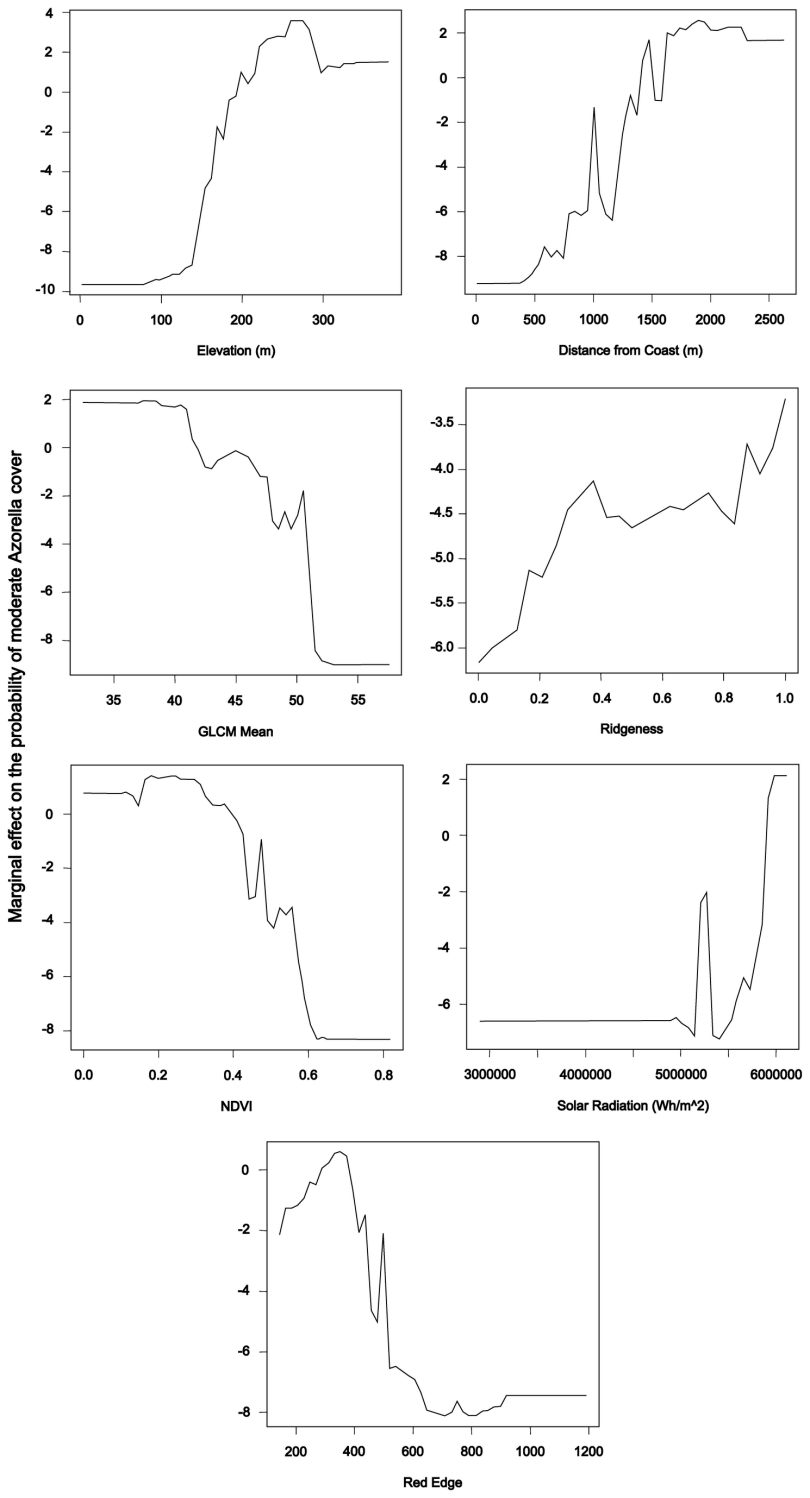
Partial dependence plots for the binary classification. Partial dependence plots for the variables selected for the pixel-based classification of Azorella presence/absence on a statistically-selected subset of input variables. The variables included were chosen on the basis of the variable importance measures. *Azorella* presence is associated with high values for elevation, distance from coast, ridgeness, and solar radiation; and with low values for GLCM mean, NDVI and red edge reflectance.

The partial dependence plots (Figure 5) showed that *Azorella* typically grows at elevations above 200 m, with NIR 1 reflectance values below 550, NDVI values below 0.55, GLCM mean values less than 50, distances from the coast greater than 600 m, the highest values for solar radiation (> 5.6 MWh/m^2^), red edge reflectance below 650, and NIR 2 reflectance less than 850 (out of 2048 DN values). On Macquarie Island, *Azorella* typically grows on the highest parts of the island (i.e. those areas with high elevation, high modelled solar radiation, and far from the coast); and is most common in feldmark. Feldmark is characterised by a mosaic of patches of plants interspersed with gravel. This results in pixels with comparatively low reflectance in the NIR and red-edge portions of the spectrum, which results in lower values for NDVI and the mean values of the GLCM matrix based on the NDVI layer. The exception is in lower-altitude grasslands where *Azorella* grows interspersed with grasses and herbs. These are the areas where the classification errors were highest, due to the difficulty in distinguishing *Azorella* from other species. The slope, shape of the terrain, topographic position, and wetness index had little effect on the classifications, as shown by low variable importance values. Excluding them from reduced classification models did not decrease the accuracy of the classifications, and often increased the accuracy marginally. Spikes in several of the partial dependence plots (e.g. Figure 5 – coast distance) appear to be caused by small clusters of plots that contain *Azorella* , but are growing in an unusual location along that environmental gradient.

Relying on the variable importance measures to select the variables for inclusion in the reduced model showed that terrain variables were most important to the classification, with the role of spectral data largely being confined to locating areas with sparse vegetation. Spearman rank correlation coefficients showed that the spectral variables incorporated into these models (GLCM mean, red edge, NIR-1, and NIR-2) were strongly correlated with NDVI (Correlation = 0.82–0.94) indicating that the major role of spectral variables in these classifications was to select areas with sparse vegetation.

### Ternary classifications

The ternary classifications resulted in very unequal class sizes in the training dataset. *Azorella* was absent from 136 of the training sites, the sparse class (< 5% cover) was found at another 44 sites, and the moderate class (> 5% cover) occurred at 21 sites. Accuracy levels remained high, however, when validated against the independent presence/absence dataset, with overall accuracies ranging between 91.6% and 92.6% (κ = 0.849–0.871; Figure 4). The pixel-based classifications minimised the variation between the accuracies of the present and absent classes (range: 89.8–96.2%) compared with the object-based classifications (range: 86.5–98.7%). There was no obvious trend in the accuracies of the classifications based on hypothesis- or statistically-driven subsets of input variables. While still high, the validation accuracies were lowest for the two classes that collectively made up the present class in the validation data (86.5–89.9%) compared with the absent class (96.2–98.7%). There was some spatial structure to the misclassifications. Of the misclassified validation sites, all but two were within 10 m of the boundary between the absent class and the two presence classes. One exception was a site containing *Azorella* , located 21–23 m from the boundary in all classifications. The other exception was site in which *Azorella* was absent but was located 81 m from the edge of the mapped *Azorella* distribution in a single classification (statistically-driven, pixel-based). This point was either correctly classified or within 10 m of the boundary for all other classifications.

**Figure 6:**
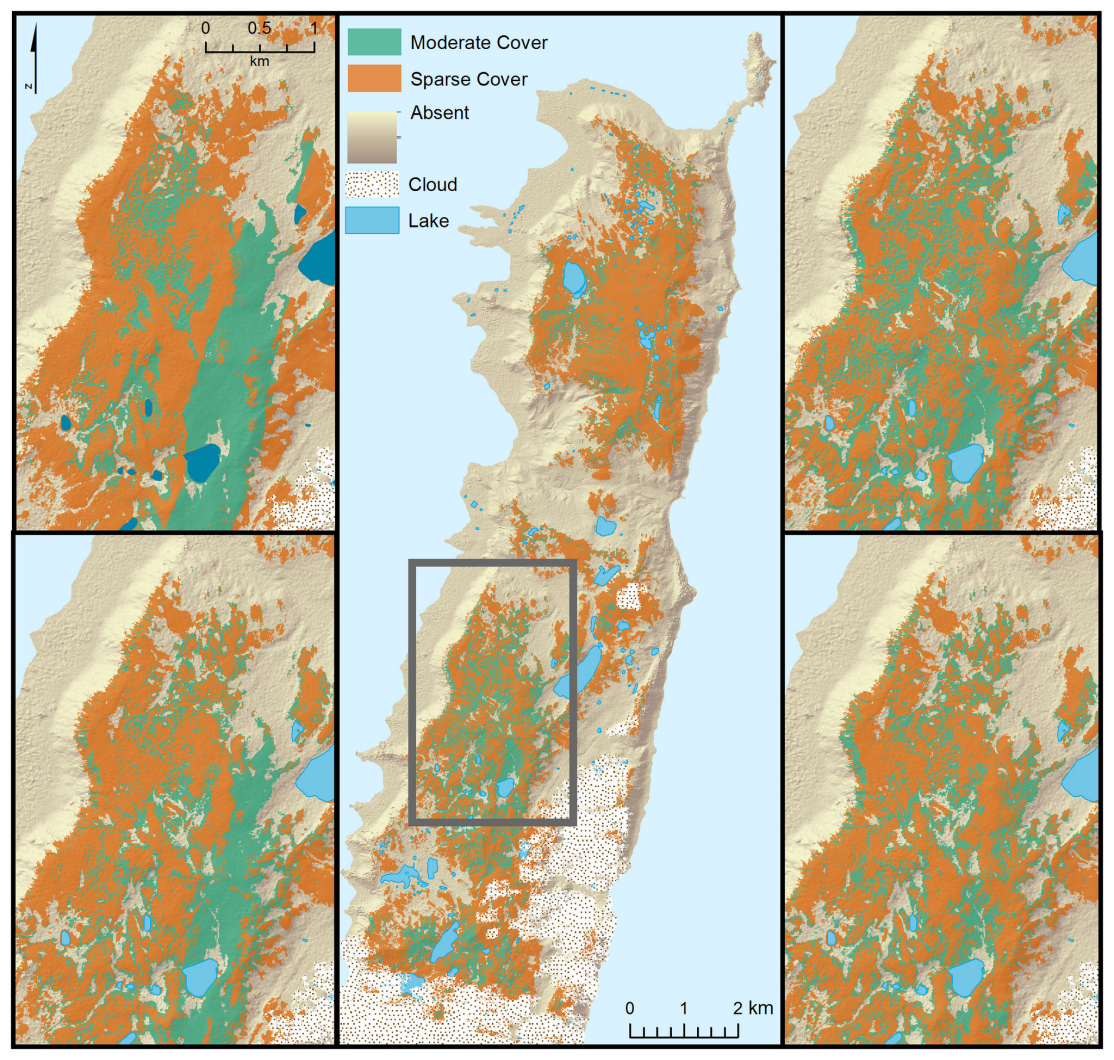
Ternary classified map of *Azorella* distribution. Predicted moderate (green) and sparse (orange) *Azorella* presence on northern Macquarie Island based on ternary classifications, showing the entire mapping region, as predicted by hypothesis-driven pixel-based classification (A). Panels B–D demonstrate the variation in maps in the central part of the mapped area, as predicted by pixel-based classification of a statistically-selected subset of input variables (B), object-based classification of a statistically-selected subset of input variables (C), pixel-based classification of a hypothesis-driven subset of input variables (D), and object-based classification of a hypothesis-driven subset of input variables (E). In general, the sparse class occurred on the highest and most exposed western-facing sites, and the moderate class occurred on east-facing flanks of the mountains, and in protected hollows, in line with current understanding of the species’ ecology, though the variation among the maps indicates that these classes could not be reliably distinguished from each other. In all classifications, both the moderate and sparse classes were clearly distinguished from the absent class.

Inspection of the resulting maps (Figure 6) showed wide variation in the predicted distributions of the moderate and sparse classes among the four classifications, but all classifications accurately and consistently predicted the distribution of the absent class. Classification accuracy can be expected to drop as the number of classes increases [40]. Here, the increased errors were concentrated in the sparse and moderate categories, in contrast to a slight decrease in errors for the absence class. Thus, it appears that there is a considerable divide between presence and absence, but it is more difficult to separate the two classes where *Azorella* is present.

In general, the maps showed that the moderate cover class was largely restricted to east-facing slopes and sheltered depressions on the plateau. Both of the statistically-driven subsets of input variables predicted a solid band of moderate *Azorella* cover running down the centre of the bottom third of the map. This appeared to be partly an artefact and largely influenced by distance from the coastline. Although there was a large amount of moderate cover *Azorella* in this area, field observations showed that it did not form a solid area of the form seen in the map. The sparse cover class included both extremely exposed west-facing slopes and areas where the feldmark meets with surrounding short grasslands, corresponding with field observations.

Inspection of the maps based on the hypothesis-driven subset of variables showed that areas of moderate *Azorella* coverage predicted with these classifications were more fragmented than in the statistically chosen set of inputs, though the moderate class was again more likely to occur on the eastern flanks of the island’s peaks and in depressions on the highest parts of the island (Figure 6). The predictions of both hypothesis-driven classifications more closely corresponded with field observations and inspection of landscape photographs than the statistically-driven classifications.

**Figure 7:**
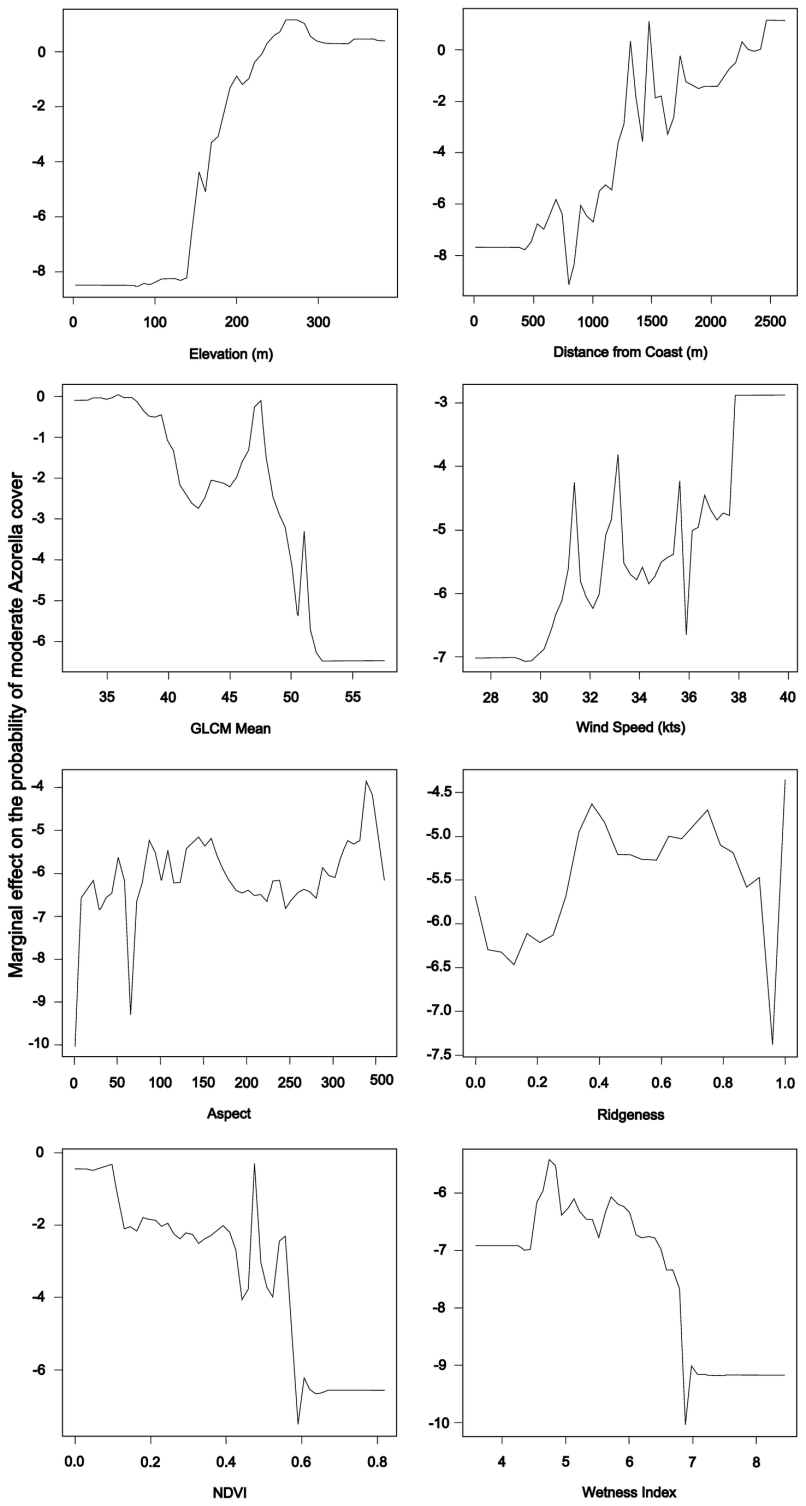
Partial dependence plots for the ternary classification. Partial dependence plots for the moderate cover class of the pixel-based classification of *Azorella* cover based on a statistically-chosen subset of input variables. The moderate cover class was associated with high values for elevation, distance from the coast, and wind speed; with low values for the GLCM mean, wetness index, and NDVI; and with mixed values for aspect and ridgeness.

The input variables chosen on the basis of variable importance measures for both the pixel- and object-based classification were elevation, NDVI, distance from the coast and GLCM mean. In addition, the pixel-based classification used topographically-deflected wind speed, aspect, ridgeness, and wetness index. The object-based classification also included NIR-1, NIR-2, red edge, solar radiation, GLCM homogeneity, and GLCM variance. This suggests that the object-based approach was more reliant on satellite variables, while the pixel-based approach depended more heavily on terrain variables. The partial dependence plots for the moderate cover class in the statistically-driven pixel-based classification (Figure 7) showed that this class was most likely at altitudes above 250 m, with a slight dip in likelihood above 300 m elevation; at sites more than 1500 m from the coast; at higher wind speeds; in areas with sparse vegetation (NDVI values below 0.6); at GLCM mean values less than 50; and in moderately dry areas (wetness index scores below seven). The relationships with aspect and ridgeness were unclear. This indicated that higher *Azorella* cover was associated with high, dry, highly exposed sites with sparse vegetation.

## Discussion

This study explored the effectiveness of several emerging tools in the field of vegetation mapping to produce high-resolution maps of the distribution of a critically endangered sub-Antarctic cushion plant. All classifications produced highly accurate maps, but in the binary classifications, the accuracies were slightly, though consistently, improved by using object-based classifications, and by using statistical measures of variable importance to choose the input variables for the classifications. These benefits disappeared in the ternary classifications. Visual inspections of the ternary classification maps indicated that the statistically-driven maps included obvious artefacts in the division between the moderate and sparse classes, though all models could reliably separate *Azorella* presence from absence.

RF classification proved to be a useful tool for mapping the distribution of *Azorella* , despite a relatively small sample size of 201 field sites; collinear input variables that made weak contributions to the classification; complex and non-linear interactions between the input variables; and noisy, non-normally distributed data. The accuracies of the classifications in this study are on a par with those found in other image classification mapping applications using a range of input data (e.g. [12,16,39]). There are three characteristics of *Azorella* that may make it better suited to mapping from satellite imagery than other species: *Azorella* has a clearly defined ecological range on the island; it is located in sparsely vegetated areas, which have a distinct spectral response; and it is often the dominant species in a structurally simple vegetation type. The strong spatial structure in the misclassification errors, with all but eight validation sites within 10 m of the boundaries for all classifications, indicates that further improvements in classification must focus on boundary detection.

One of the limitations of RF in comparison to regression-based classification methods is that it does not produce an equation with slope and intercept coefficients that can be used for direct ecological interpretation [41], though this is of secondary importance for studies such as ours in which the primary tasks are classification and production of a distribution map. The variable importance measures can be combined with visualisation tools to provide basic *post-hoc* ecological interpretation, though such interpretation must be considered hypothesis-generating rather than hypothesis-testing. The ecology of *Azorella* is already quite well described [42], but the spatial associations revealed in the partial dependence plots from these classifications quantify, for the first time, the distribution of this species in relation to landscape-scale environmental parameters. To date, discussions on the distribution of *Azorella* have been limited to general verbal descriptions, very coarse resolution maps of vegetation structure [42,43], or coarse maps based on field surveys [44]. The quantification presented here may enable future analysis of the relationships between *Azorella* dieback and topographic position as part of a multidisciplinary effort to understand the decline of this critically endangered species.

The use of GEOBIA techniques has increased in response to the availability of very high resolution satellite imagery, and researchers’ need to extract objects that were often larger than the pixels in the image [5]. To date, there has been limited testing of the relative merits of pixel- and object-based approaches when the entities being mapped do not have a granularity that obviously favours one approach or the other [14]. Here, we demonstrate very high accuracies for both approaches, when the entity being mapped ranges in size from a part of a pixel to several contiguous pixels. GEOBIA was slightly more accurate in the binary classifications, but this advantage disappeared in the ternary classifications. At such scales, there does not appear to be a clear advantage to choosing either approach.

This study highlighted some major challenges in mapping vegetation in high-latitude areas. Spatial data on environmental variables that are likely to affect plant habitat, such as soil composition, are often missing. On Macquarie Island, the presence or absence of peat is likely to be a significant predictor of the distribution of *Azorella* , but spatial data on peat distribution were not available. Furthermore, there is inherent uncertainty in attempting to collect contemporaneous field data and satellite imagery. This is particularly true in the sub-Antarctic where cloud cover often interferes with image acquisition, increasing the risk that field sites will be obscured. In this study, the exclusion of 42% of field sites left us with insufficient data to divide into training and test datasets. However, by including independent validation data from other vegetation studies we were able to mitigate these issues. Nevertheless, this approach was not optimal because the validation data could not be divided into absent, sparse and moderate classes to match the training data.

This study is part of a small but growing body of work using semi-automated image interpretation to monitor vegetation in isolated high-latitude areas. Satellite image interpretation has particular potential for examining vegetation changes where field access is limited, and this study demonstrates the high degree of accuracy possible in such areas. We are not aware of any other high-latitude remote sensing projects that have attempted to map a single species, although a few have focussed on plant communities at similar spatial resolutions, on Macquarie Island and elsewhere (e.g. [22,44,45]). In contrast, the majority of remotely sensed mapping of high latitude vegetation has examined broad vegetation types or used vegetation indices to capture general vegetation patterns [47–50]. The methods presented here demonstrate that semi-automated classification of satellite imagery can be used for vegetation mapping at much finer spatial and thematic resolution than is common in high-latitude remote sensing.

## Conclusions

Despite the limitations on the available environmental data and the reduced number of field sites that could be used to train the classifications, the classifications presented here provided highly accurate maps of *Azorella* distribution, in either two or three classes. This study has demonstrated that a critically endangered plant species can be reliably mapped using random forest classification to combine very high resolution satellite imagery and terrain modelling. Accuracies may be slightly improved by using variable importance measures to select input variables, and by using object-based classification, even for entities that are not consistently larger than the available pixel size. These maps provide a reliable baseline for monitoring expected changes in the distribution of *Azorella* on Macquarie Island. Accurate monitoring of these changes may in turn help to improve understanding of the causes of the die-back. The methods presented here are likely applicable for environmental monitoring in other areas, where a species or community occupies a relatively distinct topographic niche or is comparatively spectrally distinct.
